# Bortezomib-induced neuropathy is in part mediated by the sensitization of TRPV1 channels

**DOI:** 10.1038/s42003-023-05624-1

**Published:** 2023-12-05

**Authors:** Jared M. Sprague, Ajay S. Yekkirala, Bhagat Singh, Ivan Tochitsky, Michael Stephens, Octavio Viramontes, Jelena Ivanis, Natalia P. Biscola, Leif A. Havton, Clifford J. Woolf, Alban Latremoliere

**Affiliations:** 1 F.M. Kirby Neurobiology Center, Boston Children’s Hospital, 3 Blackfan Circle, Boston, MA USA; 2 Department of Neurobiology, Harvard Medical School, 220 Longwood Avenue, Boston, MA USA; 3 Department of Neurology, Icahn School of Medicine at Mount Sinai, New York, NY USA; 4 Department of Neuroscience, Icahn School of Medicine at Mount Sinai, New York, NY USA; 5 James J. Peters Department of Veterans Affairs Medical Center, Bronx, NY USA; 6 Department of Neurosurgery, Neurosurgery Pain Research Institute, Johns Hopkins School of Medicine, Baltimore, MD USA; 7 Department of Neuroscience, Johns Hopkins School of Medicine, Baltimore, MD USA

**Keywords:** Somatic system, Chemotherapy

## Abstract

TRPV1 is an ion channel that transduces noxious heat and chemical stimuli and is expressed in small fiber primary sensory neurons that represent almost half of skin nerve terminals. Tissue injury and inflammation result in the sensitization of TRPV1 and sustained activation of TRPV1 can lead to cellular toxicity though calcium influx. To identify signals that trigger TRPV1 sensitization after a 24-h exposure, we developed a phenotypic assay in mouse primary sensory neurons and performed an unbiased screen with a compound library of 480 diverse bioactive compounds. Chemotherapeutic agents, calcium ion deregulators and protein synthesis inhibitors were long-acting TRPV1 sensitizers. Amongst the strongest TRPV1 sensitizers were proteasome inhibitors, a class that includes bortezomib, a chemotherapeutic agent that causes small fiber neuropathy in 30–50% of patients. Prolonged exposure of bortezomib produced a TRPV1 sensitization that lasted several days and neurite retraction in vitro and histological and behavioral changes in male mice in vivo. TRPV1 knockout mice were protected from epidermal nerve fiber loss and a loss of sensory discrimination after bortezomib treatment. We conclude that long-term TRPV1 sensitization contributes to the development of bortezomib-induced neuropathy and the consequent loss of sensation, major deficits experienced by patients under this chemotherapeutic agent.

## Abstract (web summary)

An unbiased primary sensory neuron screen identifies the chemotherapeutic agent bortezomib as a potent TRPV1 sensitizer. Such long-term sensitization could lead to axonal death and sensory loss, a major symptom of bortezomib-induced neuropathy.

## Introduction

The transient receptor potential vanilloid type 1 (TRPV1) channel is a non-selective cation channel expressed by small fiber sensory neurons and plays a critical role in the detection of diverse noxious stimuli^1^. TRPV1 is a tetrameric ion channel with 6 transmembrane domains plus one pore loop^2^. The channel is directly activated by heat (~41 °C), protons, capsaicin - the pungent ingredient in chili peppers^1^ and endogenous ligands including: the endocannabinoid anandamide^3^, lipoxygenase products 12-(S)-HPETE, 15-(S)-HPETE, 5-(S)-HETE, 15-(S)-HETE, leukotriene B_4_^4^, and DAG^5^.

In addition to its direct activation by ligands, TRPV1’s activation threshold can be reduced and the current generated by the channel increased, the phenomenon of sensitization^6–11^. TRPV1 sensitization can be achieved by posttranslational modulation: PKC, PKA, PI3K, calcineurin, PDK1, and Cdk5 all phosphorylate TRPV1 at specific serine and threonine residues^12^. TRPV1 sensitization contributes to peripheral pain hypersensitivity following exposure to NGF, GDNF, IL-1β, PGE_2_, or bradykinin^13–17^. The in vitro sensitization of TRPV1 by growth factors, cytokines, arachidonic acid metabolites, lipids and peptides is generally predictive of the pain hypersensitivity producing effects of these ligands in vivo^18–20^. However, most TRPV1 sensitization studies are conducted in heterologous expression systems, which may not reflect the mechanisms responsible for in vivo sensitization produced by tissue damage and inflammation, because they do not recapitulate the natural molecular architecture of neurons and TRPV1 posttranslational changes.

TRPV1 responsiveness can be increased by agonists that reduce its inactivation time. On first exposure to capsaicin, TRPV1 desensitizes for a short period (min) to a second exposure to capsaicin^21^. This process is largely calcium-dependent, relying on the protein phosphatase calcineurin and the calcium-binding protein calmodulin to inhibit TRPV1 activation^22–24^. Reversing this short-lasting desensitization is the experimental approach most adopted as a proxy to examine TRPV1 sensitization. In vitro assays using calcium imaging^4,25^, have identified several compounds that modulate TRPV1’s responsiveness, but it is not clear if these compounds either mechanistically sensitize TRPV1 or reduce its desensitization. Since chronic inflammatory conditions are commonly associated with prolonged exposure to TRPV1-sensitizers at the site of an injury or insult, in vitro studies of a reduction in TRPV1 desensitization that are limited temporally to a period of minutes, may not fully recapitulate or be an accurate surrogate for the in vivo sensitization of the channel^26–28^.

Here, we developed a high-throughput assay of TRPV1 sensitization using mouse primary somatosensory dorsal root ganglion (DRG) neurons and screened a 480- bioactive molecule compound library (ScreenWell® ICCB Known Bioactives Library). The screen enabled us to directly assess TRPV1 sensitization, rather than changes in desensitization, and identify compounds that have actions after prolonged exposure. The use of primary somatosensory neurons in a TRPV1 sensitizer phenotypic screen instead of heterologously expressing cell lines, enabled us to uncover a novel and clinically relevant sensitizer of TRPV1, the proteasome inhibitor bortezomib. In addition, our study shows that long-term sensitization of TRPV1 promotes an excitotoxicity that leads to nerve terminal loss, a common and debilitating side effect of proteasome inhibitors.

## Results

### Screening assay

**Fig. 1:**
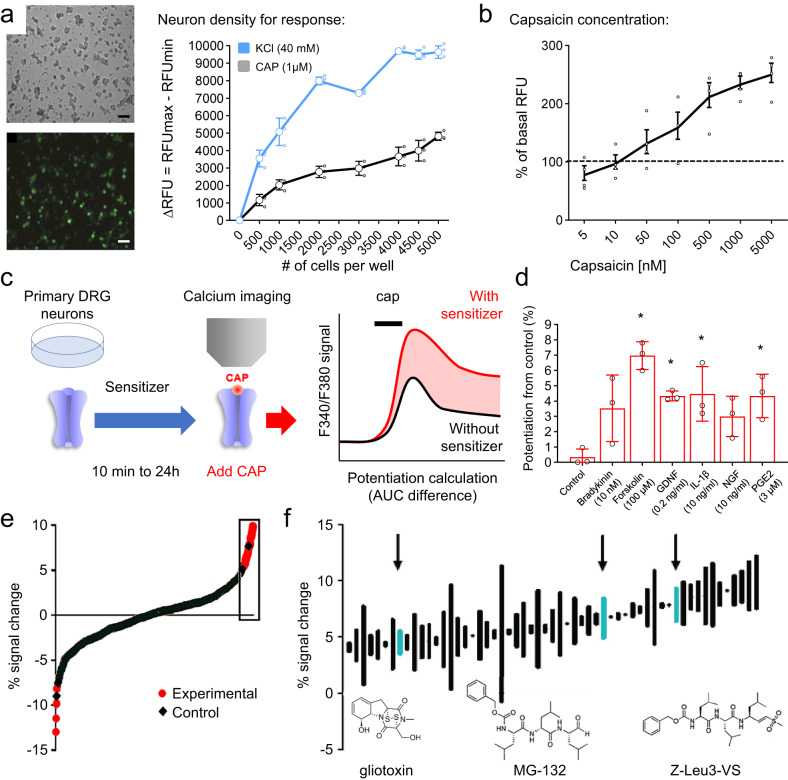
Development of an assay for TRPV1 sensitization. **a** Cells were seeded in a range of 0–6000 cells/well in 384-well plates and tested for calcium flux with KCl and capsaicin. 2000 neurons/well provided robust calcium flux responses and became the default cell density for the assay. **b** Capsaicin concentration-response curve with an EC_50_ of 121 nM. **c** Schematic representation for the assay of TRPV1 long-term sensitization from sensory primary neurons. The potentiation of TRPV1 activation is measured by calculating the AUC after capsaicin administration. **d** Functional sensitization of TRPV1 channels with a 10-min incubation of known potentiators preceding application of capsaicin (100 nM). All experiments were performed with three biological replicates. *p < 0.5 One-way ANOVA followed Dunnett’s post-hoc test, *F*^6, 14^ = 6.468. **e** Complete, ranked results of responses, each point is an average of two experiments run after 24 h of incubation with test compounds (red) or vehicle (black). % signal change represents overall change in fluorescence intensity normalized to initial intensity levels. **f** Top 8% of results showing hits that are proteasome inhibitors (Z-Leu3-VS (50 ng/mL), MG-132 (50 ng/mL), and gliotoxin (50 ng/mL)) (arrows). Bars connect two responses measured. Structures of proteasome inhibitors that cause TRPV1 sensitization. Scale bar represents 50 µm.

Mouse primary sensory neurons were used to measure calcium flux through TRPV1 channels and preserve its endogenous post-translational modifications. DRG cell culture conditions were first optimized for high throughput screening by determining the lowest cell density that provided robust and reproducible calcium ion flux signals using the FLIPR calcium 5 kit (Molecular Devices). Freshly dissociated mouse DRG neurons were seeded at increasing densities, ranging from 500 to 5000 cells/well in clear-bottom 384 well plates (Greiner) coated with laminin (1 mg/mL) (Sigma). The cells were extracted from the DRG and seeded only with native DRG cellular material, including satellite cells. No other cells were added to the culture. After 24 h in culture, the cells were treated with 1 µM capsaicin, and then afterwards with 40 mM KCl, to induce a calcium flux in TRPV1+ neurons and all neurons, respectively. The wells were imaged in an FDSS7000EX apparatus (Hamamatsu) which measures average fluorescence per well across the entire plate. Both KCl and capsaicin produced a robust calcium influx and clear, cell density-dependent responses (Fig. 1a) with high Z’-scores (KCl (*z*’ = 0.8), capsaicin (*z*’ = 0.57)) when DRG neurons were cultured at a density of 2000 cells/well. To characterize individual DRG neuronal responses we also imaged cellular changes in calcium fluorescence in response to capsaicin (1 µM) and KCl (40 mM). Almost half the neurons were activated by capsaicin (42 ± 5.3%), corresponding to ~6% of the surface area of the well (Supplementary Fig. 1a). To assess the dynamic range of calcium fluxes in this assay, we generated a concentration response curve for capsaicin. From four independent biological replicate experiments we calculated an EC_50_ of 127.2 nM (Fig. 1b), which is comparable to the EC_50_ for capsaicin in cultured cells stably expressing TRPV1^4–6^, and in cultured murine neurons^7^.

To measure the degree of TRPV1 sensitization produced by diverse compounds, we designed an assay to evaluate the effect of compound pretreatment on a subsequent response to a single (first) exposure to capsaicin (Fig. 1c). Known sensitizers of TRPV1 such as forskolin (100 µM), IL-1β (10 ng/mL), GDNF (0.2 ng/mL), and prostaglandin E_2_ (PGE_2_) (3 µM*)*^7,8^ all produced a significant sensitization (a greater calcium influx) of the response to a low concentration (100 nM) capsaicin exposure after a 10-min pre-incubation period (the same duration used in previous in vitro and in vivo studies; Fig. 1d). In addition, we could also detect whether any of the compounds screened directly produced a calcium flux upon addition (Supplementary Fig. 1b). Therefore, this assay enables screens both of the direct activation of DRG neurons, and of a subsequent TRPV1 sensitization in primary sensory neurons. We then performed a long (24 h) exposure screen using a 480-compound biologically active library (for general categories, see Supplementary Fig. 1c; for the complete list https://iccb.med.harvard.edu/biomol-iccb-l-known-bioactives-2012.

### Direct activators of sensory neurons

First, we looked for ligands in the Biomol library that directly activate sensory neurons. Of the 480 compounds, 19 produced calcium fluxes at a level similar to 1 µM capsaicin (Supplementary Fig. 1d). These included the known ion channel opener ionomycin but also novel ligands—the mitochondrial oxidative phosphorylation uncoupler (FCCP), an antibiotic (Valinomycin) and the natural product anthraquinone (Damnacanthal).

### Chemotherapeutic agents sensitize TRPV1

Next, we incubated DRG neurons with each of the 480 bioactive compounds for an extended (24-h) period of time, primarily to identify TRPV1 sensitizers or desensitizers that operate via mechanisms other than through an acute activation of protein kinases. Several ligands were found to sensitize TRPV1 after the prolonged exposure (Fig. 1e) and cancer chemotherapeutic agents were among the top such TRPV1 sensitizing agents (Supplementary Table 1). Amongst these, the three proteasome inhibitors included in the BioMol library (MG-132, Z-Leu3-VS, and gliotoxin) all sensitized TRPV1 (Fig. 1f), without causing any direct acute activation of the sensory neurons^29–31^. These results may be relevant to common adverse effects of many chemotherapeutics—pain (notably a burning sensation after administration) and peripheral neuropathy. The mechanisms responsible for chemotherapy-induced neuropathy are poorly understood and there is no effective treatment^32^. TRPV1 has been implicated in chemotherapy-induced neuropathic pain produced by paclitaxel, oxaliplatin and vincristine^33–35^, but so far, not proteasome inhibitors.

Compounds that prevented the TRPV1 sensitization are presented in Supplementary Table 2.

### Bortezomib enhances calcium flux response to capsaicin

Bortezomib (Velcade™ or PS-341) is an FDA-approved proteasome inhibitor used to treat multiple myeloma and was the first-in-class such inhibitor on the market^30^. It is a peptide boronic acid, analogous to a common class of protease inhibitors, aldehyde peptides^36^ and inhibits the 26S proteasome complex with high specificity and affinity (K_i_ of 0.6 nM). The proteasome complex is responsible for the degradation of many cell-cycle regulatory proteins including cyclins, tumor suppressor genes (e.g., p53), oncogenes (e.g., c-myc), IκB, p130, and enzymes such as topoisomerases. A buildup of these products leads to apoptotic activation. Bortezomib-induced peripheral neuropathy occurs in 30–50% of patients^37^.

**Fig. 2:**
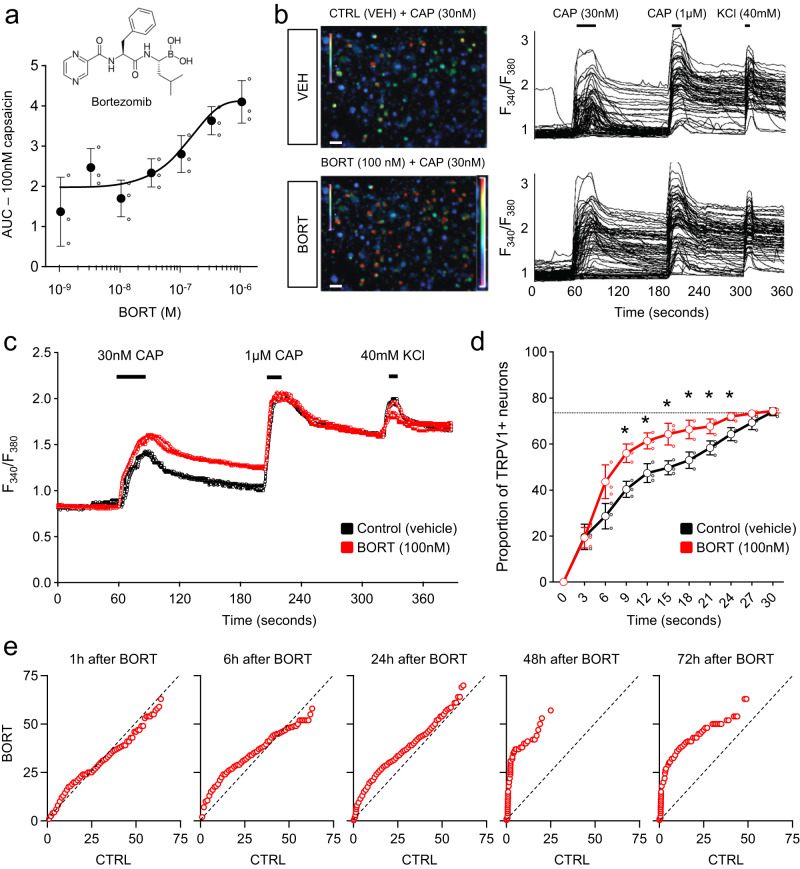
Unbiased screen uncovers proteasome bortezomib as a long-term TRPV1 sensitizer. **a** Bortezomib concentration-dependent increase in TRPV1 sensitization. **b** Left, representative images of calcium flux in sensory neurons in response to capsaicin 30 nM (CAPS) after treatment with SES buffer (top) or after 24 h pre-incubation of bortezomib 100 nM (bottom). Right, individual fura-2 based calcium tracings of DRG neurons after a 24 h treatment with a single application of SES buffer (top) or treated with 100 nM bortezomib for 24 h (bottom). **c** Average tracing for all TRPV1+ cells after incubation of SES buffer or bortezomib, includes a low dose of capsaicin (30 nM) followed by a higher dose of capsaicin (1 µM) to activate any remaining capsaicin-sensitive neurons in vitro, and then finally a high dose of KCl (40 mM) to define the dish population of responsive neurons. Three replicates are shown per condition. **d** Proportion of DRG neurons responsive after exposure to 30 nM capsaicin after bortezomib treatment. Mean and standard error from three different trials. Dashed line represents maximum proportion of TRPV1+ neurons responsiveness. *: *p* < 0.5 Two-way ANOVA followed by Sidak’s post-hoc test. *F*^10, 40^ = 4.751. **e** Quantile-quantile analysis of the number of neurons with TRPV1 sensitization after various times of pre-incubation of bortezomib. The X-axis represents the unitless area under the curve (AUC) response of control cells to a first dose of 30 nM capsaicin compared with the AUC of an interpolated matching quantile experimental cell exposed to a first dose of 30 nM capsaicin following treatment with 100 nM bortezomib for the times indicated. The dotted line with a slope of 1 indicates the null-hypothesis, where the two populations of cells behave similarly upon activation by capsaicin. Bending towards the Y-axis indicates stronger responses to capsaicin among cells pre-treated with bortezomib. Scale bar represents 40 µm.

Due to the strong sensitization of TRPV1 by all three proteasome inhibitors in the screen, as well as the high incidence of neuropathy in bortezomib-treated patients, we tested if this drug has a similar effect. Bortezomib was added to cultured mouse DRG neurons immediately after plating, and TRPV1 sensitivity to a low-concentration capsaicin challenge (30 nM) assessed 24 h later s in the primary screen. Bortezomib produced a concentration-dependent long-term sensitization of TRPV1 in our assay (Fig. 2a) and in microscope-based calcium imaging with the ratiometric dye, Fura-2 (Fig. 2b,c). Next, we measured the amplitude and kinetics of the capsaicin response, as well as the number of responding cells. For these experiments we used a concentration of 100 nM bortezomib, equivalent to early plasma concentrations following administration of the drug in patients^38^. Pre-incubation with bortezomib caused an increased response to a sub-saturating concentration of capsaicin (30 nM) compared to cells pre-incubated with Hank’s Balanced Salt Solution (HBSS; Fig. 2c). The overall response to a subsequent application of saturating dose of capsaicin (1 µM) was similar between bortezomib or HBSS-treated neurons (Fig. 2c). Pre-treatment with bortezomib caused a greater proportion of neurons to be activated faster by capsaicin (30 nM). The final proportion of neurons activated was not, however, changed (Fig. 2d).

Next, we modified the bortezomib pre-treatment duration to determine the onset of the effect of TRPV1 sensitization. Longer durations correlated with an increasing effect across 1-h, 6-h, 24-h, 48-h, and 72-h time frames. While the maximum effect was detected after a 48 h pre-incubation of bortezomib, the effects were observed as soon as after 1 h pre-incubation (Fig. 2e). This sensitization was not likely caused by an increase in TRPV1 levels, as indicated by unaffected transcripts levels (Supplementary Fig. 2a), the same response to 1 µM capsaicin (Fig. 2c) and the rapid sensitization onset (between 1 and 6 h; Fig. 2e). Altogether, these results suggest that bortezomib causes a direct sensitization of TRPV1 rather than a ‘rescue from desensitization’ or an increase in TRPV1 expression.

### Bortezomib enhances electrophysiological responses to capsaicin

**Fig. 3:**
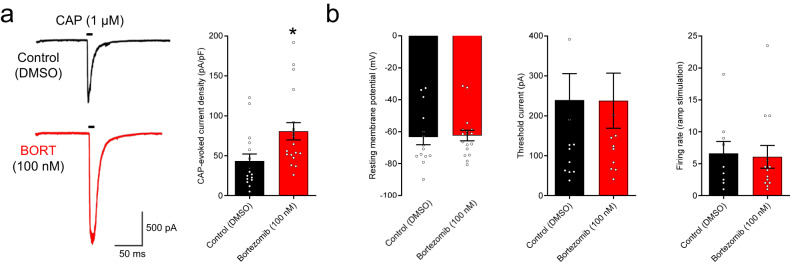
Bortezomib enhances capsaicin-evoked currents. **a** Left, voltage clamp traces of current evoked by perfusion of 1 µM capsaicin from a naive mouse DRG neuron and a neuron treated with 100 nM bortezomib for 24 h. These cells are also capsaicin-naïve prior to their 1 µM dose of capsaicin. Right, capsaicin evoked current density in naïve (black, 44 ± 9 pA/pF, *n* = 16) and bortezomib treated DRG neurons (red, 81 ± 11 pA/pF, *n* = 19, *p* < 0.05, Student’s *t* test, *t* = 2.605, df = 33). **b** Left, resting membrane potential in naïve and bortezomib treated capsaicin sensitive (TRPV1+) mouse DRG neurons (*n* = 14–20 for each group, *p* > 0.05 for all). Middle, minimum (threshold) current injection necessary to elicit an action potential in naïve and bortezomib treated capsaicin sensitive (TRPV1+) mouse DRG neurons (*n* = 14–20 for each group, *p* > 0.05 for all). Right, firing rate (Hz) in response to a 2 nA ramp current injection stimulus in naïve and bortezomib treated capsaicin sensitive (TRPV1+) mouse DRG neurons (*n* = 14–20 for each group, *p* > 0.05 for all). Data presented as mean ± SEM. Dots represent individual cells.

To test formally whether bortezomib specifically sensitizes TRPV1 channels, we recorded capsaicin evoked TRPV1 currents in DRG neurons. We used freshly dissociated mouse DRG neurons incubated with 100 nM bortezomib for 24 h then washed off the bortezomib and performed voltage clamp recordings. We found that capsaicin (1 μM)-evoked currents were larger in bortezomib pretreated DRG neurons compared to untreated DRG neurons (Fig. 3a). After normalizing the currents for cell size (capacitance), capsaicin-evoked current density was 84% higher in bortezomib treated neurons (*n* = 16–19, *p* = 0.012, Student’s *t* test). Bortezomib, however, did not change the resting membrane potential, the threshold current for action potential firing, or the firing rate produced in response to a 2 nA ramp stimulus (Fig. 3b). These results suggest that bortezomib does not cause a general increase in sensory neuron excitability but specifically sensitizes TRPV1 over many hours. In support of this, reports of a neuronal hyperexcitability in bortezomib-treated human patients^39^ was only observed 3–5 weeks after the initiation of treatment, suggesting that changes in neuronal excitability may take longer to emerge than TRPV1 sensitization.

### Bortezomib-induced neurotoxicity

**Fig. 4:**
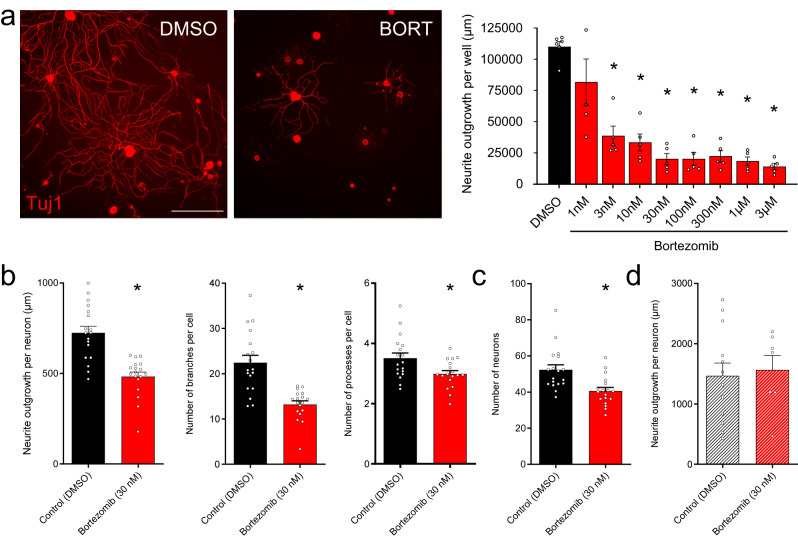
Bortezomib-induced neurotoxicity. **a** Representative images of primary DRG neurons stained with Tuj1 after treatment with DMSO or Bortezomib (30 nM). Right, dose response curve of bortezomib action on neurite outgrowth measured by well indicating significant loss of neurites after 24 h of treatment. *p* < 0.05 compared to DMSO, one-way ANOVA followed by Dunnett’s multiple comparisons test, *F*^8, 37^ = 20.58. **b** Decrease in average neurite outgrowth per neuron, number of branches per neurite and number of processes per neuron with bortezomib treatment. *, *p* < 0.05, Student’s *T* test (neurite outgrowth: *t* = 5.468, df = 34; number of branches: *t* = 5.131, df = 34; number of processes: *t* = 2.595, df = 34). **c** Number of neurons estimated after incubation with DMSO or bortezomib 30 mM. *, *p* < 0.05, Student’s T test (*t* = 3.486, df = 34). **d** Mean neurite outgrowth per neuron from TRPV1 KO mice treated with DMSO or bortezomib 30 mM. *, Scale bar: 100 microns. Data presented as mean ± SEM. Dots represent individual neurons.

We treated mouse DRG neurons with different doses of bortezomib (3 nM–10 µM) in vitro and analyzed neurite outgrowth. Analyzing the overall neurite outgrowth per well we found a dose dependent decrease starting at 3 nM bortezomib (*p* < 0.001) with a maximal response observed at 30 nM (Fig. 4a). At this dose, bortezomib co-incubation reduced average neurite length, the number of branches per neurite and the number of processes per neuron (Fig. 4b) and caused neuronal death (Fig. 4c).

Since bortezomib sensitizes TRPV1 and this channel has been linked to axonal toxicity, we speculated that long term TRPV1 sensitization may contribute to bortezomib-induced neurotoxicity. To test this hypothesis, we treated cultured DRG neurons from mice lacking TRPV1 channel (TRPV1 KO) with bortezomib or vehicle, and found that TRPV1 KO DRG neurons incubated with bortezomib do not show any defect in neurite outgrowth or neuronal death (Fig. 4d).

### Neuroprotection in mice lacking TRPV1 in vivo

**Fig. 5:**
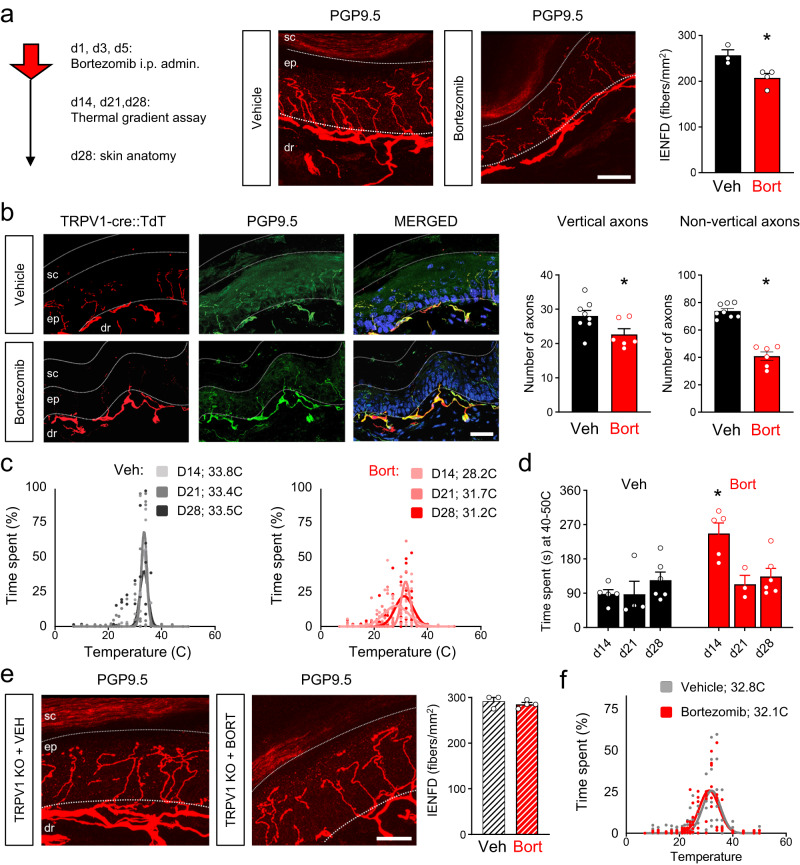
TRPV1 KO mice are protected against bortezomib-induced neurotoxicity and loss of sensation in vivo. **a** Top, In vivo dosing and behavioral measurement and anatomical studies schedule. Bottom, intraepidermal nerve fiber density (IENFD) assessed by PGP95 staining in WT mice treated with bortezomib treatment or vehicle. *: *p* < 0.05, Student’s *T* test (*t* = 3.267; df = 5). **b** IENFD assessed in TRPV1-cre::TdT mice to label TRPV1-lineage sensory neurons. In red, TdT signal, in green PGP9.5 staining and in blue DAPI signal. SC: stratum corneum, EP: epidermis, DR: dermis. Quantification classified vertical and non-vertical axons in the skin section. *: *p* < 0.05, Student’s *T* test (*t* = 2.287; df = 12 for vertical and *t* = 9.666; df = 12 for non-vertical) **c** Time spent in different temperature zones during the last 30 min of the thermal gradient assay in mice treated with saline and bortezomib and tested 14, 21, and 28 days after cessation of treatment. Preferred temperature is calculated by the best-fitted Gaussian curve and peak is indicated on top in °C. 95% confidence interval for temperature preference (in °C): Vehd14: 33.05 to 33.7; Vehd21: 33.03 to 33.71; Vehd28: 33.31 to 33.8; Bortd14: 27 to 29.7; Bortd21: 30.26 to 32.13 and Bortd28: 30.31 to 32.85). **d** Time spent in a zone of 40–50 °C (noxious temperature) in saline and bortezomib treated mice tested 14, 21, and 28 days after cessation of treatment. *: *p* < 0.05 compared to veh d14 (*, *p* < 0.05 Sidak’s test, *t* = 5.347, df = 5.352). **e** IENFD in TRPV1 KO mice treated with saline or bortezomib. **f** Time spent in different temperature zones during the last 30 min of the thermal gradient assay in TRPV1 KO mice treated with saline or bortezomib and tested 14 days after cessation of treatment. Preferred temperature is calculated by the best-fitted Gaussian curve and peak is indicated on top in °C. 95% confidence interval for temperature preference (in °C): Veh: 31.17–32.92; Bort: 30.57–32.71). For anatomy, *n* = 3–6 mice per group. For behavior: 5–6 mice per group. Data presented as mean ± SEM. Dots represent individual animals. Scale bar represents 50 µm.

Next, we tested if TRPV1 is involved in bortezomib-induced neuropathy in vivo. We injected bortezomib intraperitoneally (3 injections of 1 mg/kg on alternate days (day 1, 3, and 5) and harvested skin 2 weeks after cessation of treatment (Fig. 5a). Intraepidermal nerve fiber density (IENFD) analysis using the pan-neuronal marker PGP9.5 in WT mice showed a significant reduction of fiber density in mice treated with bortezomib (Fig. 5a). A similar analysis in reporter mice expressing TdTomato under the TRPV1 promoter (TRPV1::TdT mice) confirmed that TRPV1-lineage fibers were particularly affected, especially the most superficial axons spreading along the skin (non-vertical axons; Fig. 5b, Supplementary Fig. 2). To assess the consequences of this loss of sensory fibers on sensory function after bortezomib treatment, we used a thermal gradient apparatus, where mice can freely explore a chamber for 90 min and be exposed to both noxious and innocuous temperatures ranging from 7 °C to 55 °C and choose a temperature where they feel most comfortable^40^. While mice injected with saline showed a clear thermal preference around 33.6 ± 0.1 °C at all time points, bortezomib-treated animals displayed a preference towards colder areas (30.5 ± 1.12 °C; Fig. 5c; Supplementary Fig. 3c). In addition, bortezomib-treated mice did not in contrast to vehicle-treated animals, settle into a narrow preferred thermal zone, suggesting a deficit in innocuous sensory discrimination (Fig. 5c; Supplementary Fig. 3c). Avoidance of noxious heat (>40 °C) was impaired 1 week after cessation of bortezomib treatment, and this returned to normal values 1 week later, suggesting either a partial return of nociceptor innervation or the sensitization of the remaining terminals (Fig. 5d).

TRPV1 KO mice had a similar intraepidermal nerve fiber density as WT mice at baseline, and bortezomib treatment did not cause a significant decrease in these animals (Fig. 5e), suggesting that a lack of TRPV1 on exposure to bortezomib is neuroprotective. In support of this anatomical observation, TRPV1 KO mice treated with bortezomib did not show a thermal place preference defect compared to vehicle-treated animals (Fig. 5f, Supplementary Fig. 3d).

## Discussion

TRPV1 channel sensitization triggered by endogenous ligands generated under inflammatory conditions plays an important role in pain hypersensitivity and thermal regulation^4, 26–28,41,42^. Given the importance of this pain-enhancing mechanism, we sought to identify novel ligands and pathways that sensitize TRPV1. We aimed to identify small molecules that only begin to sensitize nociceptor neurons over a period of hours, similar to the time course of post-injury pain in humans. Previous in vitro studies of TRPV1 sensitization^26–28^ have several drawbacks: they generally only measure short-term sensitization over a period of minutes, most are conducted in heterologous expression systems, and often measure a reduction in desensitization rather than direct sensitization. These short-term in vitro studies of TRPV1 sensitization may not, therefore, reflect the totality of mechanisms responsible for the sensitization in vivo arising in the setting of tissue damage and inflammation. Heterologous systems using cell lines such as TRPV1-expressing HEK cells cannot accurately or fully recapitulate the natural molecular architecture of neurons, receptors, and pathways, both in their native state and under conditions where post-translational changes occur. While a decrease in desensitization reflects the development of some forms of acute sensitization, this may not be true for the situation of delayed sensitization. To deal with these challenging issues, we developed a novel phenotypic assay to identify TRPV1 sensitizers. This assay has several advantages: it is suitable for a 384 well plate format and can be used to conduct unbiased small molecule or peptide screens. It allows for the study of long-term/delayed sensitization over hours or days and enables measurement of the direct sensitization of TRPV1 in mouse primary neurons. This unbiased assay identified after a 24-h exposure, novel sensitizers, including cancer chemotherapeutic agents and proteasome inhibitors, a class of compounds not previously reported to activate or sensitize TRPV1.

Chemotherapy-induced peripheral neuropathy (CIPN) is a dose limiting and debilitating side effect of cancer chemotherapy affecting 30–50% of all patients undergoing such treatment^43^. The symptoms can be severe and include burning pain soon after treatment and chronic pain long after treatment has ceased^44^. One major complaint from patients undergoing chemotherapy is a loss of sensation which is due to a reduced epidermal innervation, typically most prominent in the extremities in a glove and stocking fashion. CIPN is caused by many classes of chemotherapeutic agents including vinca alkaloids (vincristine, vinblastine), platinum-based compounds (cisplatin, oxaliplatin), taxanes (paclitaxel, docetaxel), and proteasome inhibitors (bortezomib), regardless of their very different molecular mechanisms of action. Our discovery that TRPV1 function is enhanced by exposure to proteasome inhibitors led us to test bortezomib, the prototypic proteasome inhibitor used in the clinic, and it proved also to be a strong sensitizer of TRPV1, both in our primary assay, and in secondary assays using calcium imaging and electrophysiology.

Bortezomib produces varying degrees of sensory neuropathy in about half of treated patients, with one out of six patients developing severe neuropathy^45,46^ and about 30% of patients a neuropathy that does not recover^47,48^. We exposed DRG neurons to various doses of bortezomib and found potent toxic effects on neuronal outgrowth and cell death. This neurotoxic effect was replicated in vivo with a significant reduction in intraepidermal nerve fiber density in mice treated with bortezomib, and an associated loss of thermal sensory discrimination. High doses of bortezomib have been proposed to cause neuronal death acting via SARM1 and downstream nicotinamide adenine dinucleotide loss^49,50^. Interestingly, subcutaneous rather than IV administration of bortezomib has been shown to mitigate the high peak of plasma drug levels, but this regimen is still associated with the development of sensory neuropathy^51^. Our data suggest that this neurotoxicity is mediated by a long-term sensitization of TRPV1, as this channel is strongly expressed in skin axon terminals where nerve terminal retraction occurs and TRPV1 null mice were much less susceptible to bortezomib neurotoxicity compared to littermates. Our results show that the long-term sensitization of TRPV1 channels by bortezomib does not change their activation threshold, arguing against a change in local tissue temperature as a contributing factor for the channel’s opening in vivo. Since most patients complain of a burning sensation at the time of bortezomib administration, it is possible that this compound directly or indirectly causes a transient activation of TRPV1 channels. In addition, it is likely that the axonal degeneration process caused by bortezomib releases several proinflammatory mediators, thereby causing an ongoing process where dying axons can activate nearby sensitized TRPV1 channels. TRPV1 overactivity leads to a calcium overload in axons which causes axonal degeneration by activating calcium sensitive enzymes like calpain^52,53^ as does activation of SARM1 which can cause mitochondrial dysfunction^50^. Such a neurotoxic calcium overload can be caused by strong capsaicin-evoked currents^54,55^, which can be enhanced by an increase in TRPV1 protein levels at the membrane^56^ due to impaired membrane trafficking^57^ and/or, as we propose here, by the direct, long-term sensitization of TRPV1 channels. These multiple mechanisms could act together and explain the toxic effects of bortezomib on TRPV1-expressing axons.

Using a novel phenotypic screen, our study has identified that exposure to proteasome inhibitors leads to a TRPV1 sensitization that only develops after hours of treatment exposure. This phenotypic screen can now be extended to examine other bioactive compounds, especially chemotherapeutic agents, and may help identify those with a lower risk of CIPN. Our data suggests that blocking long-term TRPV1 sensitization may have a role in preventing development of bortezomib induced CIPN, at least in nociceptors, which should not impact the cancer chemotherapeutic activity of the agent.

## Methods

### Dissection and culture of DRG neurons

Male adult C57Bl/6j mice (8–10 wks; 20–25 g) were purchased from Jackson Laboratories and housed in the animal facilities of Children’s Hospital Boston on a 12-h alternating light-dark cycle. Animals for imaging were dissected after 7 weeks of age similarly to the procedures outlined previously^58^. After CO_2_ asphyxiation and cervical translocation, and following spinal laminectomy, the left and right DRG from the whole spine were removed and placed in 4 °C HBSS (Life Technologies). After the DRG were collected and spun down for 5 min at 1000 rpm (150 g), they were placed in a collagenase/dispase solution (3 mg/mL dispase II and 1 mg/mL collagenase A, Roche Applied Science) and allowed to incubate at 37 °C for 90 min. After incubation the cells were washed in Dulbecco’s Modified Eagle’s Medium (DMEM, Life Technologies), fortified with 4.5 g/L D-glucose, L-glutamine, 110 mg/L sodium pyruvate, 10% fetal bovine serum (Life Technologies), penicillin (500 U/mL, cellgro), and streptomycin (500 µg/mL, cellgro). DNAse (125 U/mL, Sigma) was then added and the solution was triturated using successively smaller caliber flame-polished pasteur pipettes. This solution was gently layered onto a bovine serum albumin gradient (10% albumin from bovine serum, Sigma in PBS, Life Technologies) and spun at 150 g for 12 min. After removal of the supernatant, the cells were washed again in DMEM, suspended in neurobasal medium (Life Technologies) supplemented with L-glutamine (20 mM, Life Technologies), B-27 supplement (Life Technologies), penicillin (500 U/mL, cellgro), and streptomycin (500 µg/mL, cellgro) and then plated onto Poly-D-Lysine (PDL) and laminin-treated (1 mg/mL, Sigma) 15 mm glass-bottom dishes (MatTek Corporation), or placed into laminin-treated 384-well optical plates (Greiner) and then placed in an incubator at 37 °C (5% CO_2_) overnight. Plating density in the 15 mm optical plates was between 150–250 cells/µL, while in the 384-well optical plates was 80–120 cells/µL.

### High throughput calcium imaging

Cells were plated at 2000 cells/25 µL/well in neurobasal medium (Life Technologies) in laminin-coated (1 mg/mL, Sigma) borosilicate glass-bottomed and black polystyrene cased 384-well plates (Sensoplate, Greiner). Within 3 h of plating the cells, a 2.5 µL drop of the 384-well screening plate (ICCB, Screen-Well® Bioactive Molecules, plates 3402 and 3403) was added using the FDSS7000EX (Hamamatsu). After overnight incubation at 37 °C with 5% CO_2_, the wells were then loaded with 20 µL of FLIPR 5 calcium dye (Molecular Devices). We preloaded all ligand solutions at 5× concentrations in individual v-shaped conical-bottomed 384-well polypropylene plates (Greiner) when we custom prepared the ligand plates. Library-based ligand plates were purchased from the Institute of Chemistry and Cell Biology (ICCB, Harvard Medical School) screening facility. Solutions were added following mechanical trituration from stages 1 and 2 at 10 and 12 µL, and 10 µL/s and 12 µL/s respectively. Solutions were added at a height of 2.4 mm from the bottom of the assay plate. Primarily, the drop period with the following 20–30 s was sampled at 2.5 Hz, otherwise samples were taken at 0.33 Hz. The camera used is a digital CCD camera C9100-13 (Hamamatsu) with an exposure time of 200 ms with 2 × 2 binning. The light source is a LightningCure™ LC8 (Hamamatsu) bulb that was passed through a 472 nm excitation filter and collected at 540 nm. Solutions of capsaicin (end concentration of 100 nM - drop of 10 µL) and KCl (end concentration 5 mM for sub-maximal and 40 mM for maximal with drop of 12 µL) or HBSS (vehicle for all solutions) were added at various times. The responses were measured as relative fluorescence units, initially captured using FDSS7000EX/uCell software and analyzed on Excel (Microsoft) for area under the curve (AUC), plotted on Prism analysis and visualization software (Graphpad). Data points are collected as relative fluorescence and then are modified to ratio values post-collection. The first frame of each well equals the baseline, and all following responses are calculated as a ratio of the initial fluorescence (Ratio). Percent change of response was calculated as:

1 $$\frac{{F}_{x}-{F}_{y}}{{F}_{y}}$$

Where ${F}_{x}$ is the fluorescence signal of all accumulated signal (AUC) of the treated well, and ${F}_{y}$ is the AUC fluorescence ratio signal of the untreated well, but still activated with capsaicin. In some experiments additional controls were used to calculate the actual % sensitization or desensitization. These required treated controls that, instead of capsaicin agonism following treatment were given vehicle control (HBSS). % sensitization is calculated by first subtracting the treated negative control (vehicle following treatment) from the treated and challenged wells and is referred to as the ΔRatio. The ΔRatio is used as in Formula^1^ to calculate % change as an actual sensitization or desensitization of response.

### Compound library

The ScreenWell™ ICCB Known Bioreactives Library (BioMol) was purchased through the Assay Development and Screening Facility Core at Boston Children’s Hospital who worked with the Institute of Chemistry and Cell Biology at Harvard Medical School (Boston) to formulate the plate. The BioMol library is a 480-compound library of well-annotated chemicals with diverse topologies and mechanisms of action. The complete list of compounds can be found here: https://iccb.med.harvard.edu/biomol-iccb-l-known-bioactives-2012. The daughter library plate was formed by adding a 30 nL pin drop to 300 µL HBSS for a 10000-fold dilution. This library was kept at 4 °C for 2 weeks and used within that time. Many of the compounds in the Screen-Well® ICCB Known Bioactives (BioMol) Library activate specific signaling pathways and receptors as well-suited experimental compounds in addition to including a large proportion of compounds that are used in the clinic. Although this library is small by industry-standards, it offers a wide and diverse set of topologies and targets, well suited for an experiment in primary sensory neurons^59^.

### Calcium imaging

DRG neurons were imaged on a Nikon Ti Eclipse inverted microscope. Fura-2, AM (Life Technologies) was loaded into the neurons for 30 min (room temperature, 4 µg/mL). After washing with standard extracellular solution (Boston BioProducts), the cells were imaged using a QImaging EXi Aqua cooled camera and data was collected and analyzed using NIS Elements software (AR 3.10). Neurons were selectively exposed to various solutions via a gravity-assisted perfusion system. Responses were included if they were 20% greater than baseline. The perfusion rate of the solutions was 0.6 mL min^−1^ and the pencil tip was 150 µm away from the field of view.

### RNA extraction

Cells were cultured under standard plating and imaging conditions. After treatment or control, the media is removed and 500 µL TRIzol® (Life Technologies) is added and cells are scraped off and mixed well, incubating at RT for 5 min before adding to a −80 °C freezer for at least 1 h. After thawing, we add 100 µL chloroform to each sample, shake vigorously for 15 s, incubate at RT for 3 min, and then spin at 4 °C at 12000 g. The aqueous, chloroform phase we transfer to a fresh tube on ice and. 1 µL of GlycoBlue™ (Life Technologies) is added to each sample, then 250 µL of isopropanol is added, and then incubated at least an hour at −80 °C. After thawing, the samples are centrifuged at 12,000 *g* at 4 °C for 20 min. The supernatant is removed and discarded and then the samples are washed with 500 µL 75% cold ethanol and then centrifuged at 7400 *g* at 4 °C for 15 min. The ethanol is removed and the pellet allowed to air dry. Each pellet is resuspended in 12.5 µL nuclease-free water, incubated at RT for 3 min, placed on ice and quantified for RNA content using a Nanodrop spectrophotometer.

### cDNA synthesis and qPCR

cDNA is prepared using the SuperScript® Vilo cDNA synthesis kit (Life Technologies) according to manufacturer’s instructions. qPCR is performed using the TaqMan® probe set for mouse TRPV1 (Mm01246302_m1) and GAPDH (Mm03302249_g1) as control, following manufacturer’s instructions (Life Technologies). Fold changes were calculated using the comparative C_T_ method^60^.

### Electrophysiology

DRG neurons were cultured as previously described^61^. DRGs were dissected from adult C57Bl/6j mice (12 weeks, Jackson Laboratories) into Hank’s balanced salt solution (HBSS) (Life Technologies). DRGs were dissociated in 1 μg ml^−1^ collagenase A plus 2.4 U ml^−1^ dispase II (enzymes, Roche Applied Sciences) in HEPES-buffered saline (Sigma) for 90 min at 37 °C and then triturated down to single-cell level using glass Pasteur pipettes of decreasing size. DRGs were then centrifuged over a 10% BSA gradient and plated on laminin-coated cell culture dishes (Sigma). DRGs were cultured 24 h in B27-supplemented neurobasal-A medium plus penicillin/streptomycin (Life Technologies) with or without 100 nM bortezomib. After 24 h in culture, bortezomib was washed off and electrophysiological recordings performed.

Whole-cell current-clamp and voltage-clamp recordings were performed using a Axopatch 200A amplifier (Molecular Devices) at 25 °C. Data were sampled at 20 kHz and digitized with a Digidata 1440 A A/D interface and recorded using pCLAMP 10 software (Molecular Devices). Data were low-pass filtered at 2 kHz. Patch pipettes were pulled from borosilicate glass capillaries on a Sutter Instruments P-97 puller and had resistances of 2–4 MΩ. Series resistance was 5–10 MΩ and compensated by at least 80%. Responses to capsaicin (1 μM) were measured in voltage clamp at a holding potential of −80 mV. A 2 nA ramp stimulus was delivered over 1 s in current clamp mode to measure DRG excitability. The external solution for electrophysiological recordings consisted of (in mM): 140 NaCl, 3 KCl, 1 MgCl_2_, 1 CaCl_2_, 10 glucose and 10 HEPES, pH 7.3. The internal pipette solution consisted of (in mM) 140 KCl, 0.5 EGTA, 5 HEPES and 3 Mg-ATP, pH 7.3.

### Bortezomib treatment

All procedures were approved by the Boston Children’s Hospital’s Institutional Animal Care and Use Committee and we have complied with all relevant ethical regulations for animal testing. Bortezomib was dissolved in a 10% dextran solution in saline. Wild-type C57Bl/6j mice and TRPV1 KO mice were treated intraperitoneally with either Bortezomib (1 mg/kg) dissolved in saline with 10% dextran or vehicle (10% dextran in saline) for three times (day 1, 3 and 5). Mice underwent sensory testing (thermal gradient assay), at baseline before injections and at day 14, 21 and 28 after injections. All animals were socially housed on a 12 h light dark cycle with water and food ad libitum. Experimenter was blinded to the identity of the groups for all behavioral measurements and measurements were conducted on the same days of week in the same room to limit biases and variability.

### Thermal gradient

Animals were habituated individually for 3 days prior to behavioral experiments. One baseline measurement was made prior to treatment. A continuous temperature gradient (7–50 °C) was established along a metallic base plate on which the mice walked freely while being video-recorded from above (Bioseb, France). After an exploration period, the mouse showed a distinct preference, indicating the most comfortable temperature range. Data are presented by time spent on zones set at specific temperatures (7, 10, 12, 15, 17, 19, 21, 22, 23, 24, 25, 27, 30, 32, 34, 36, 40, 44, 48, 50 °C). For each condition, the best-fitted Gaussian curve was applied for the last 30 min of the trial and the preferred temperature was determined as the peak. The sensory discrimination index was estimated by determining the confidence interval during these last 30 min. Statistical comparisons were carried out on the calculated preferred temperatures. Each run lasted 1.5 h, and two mice were simultaneously recorded in separate corridors. Mice were tested weekly up to day 28 after the first injection.

### Intraepidermal nerve fiber density (IENFD)

IENFD of skin tissue samples taken from the left hind foot pad of each mouse was analyzed 28 days after the first injection. The samples were fixed in OCT and cryopreserved at −80 °C. Tissues were cut in 25 μm sections and mounted onto slides. Samples were then immuno-stained using PGP 9.5 primary antibody (1:1000 Millipore-sigma # AB5898; in 1X PBS) followed by Alexa Fluor 568 (Thermofisher; #A-11075) or Alexa Fluor 488 (Thermofisher; #A-11073) secondary antibodies (1:500 dilutions in 1X PBS) and mounted using Vectashield mounting medium containing DAPI (#H-1200). Samples were washed with 1X PBS and TritonX100 was used as a detergent. Sections were imaged using confocal microscopy at 40× magnification. Once the epidermal border of a sample was identified, six consecutive images were taken per sample. Five samples were imaged for each mouse for a total of 30 images. The total number of nerve fibers penetrating the epidermis at a right angle (emerging from dermis and entering to epidermis) and other nerve fibers within the epidermis were analyzed by dividing the number of fibers per mm^2^ of area.

### Neurite outgrowth analysis

Primary DRG sensory neurons cultures and neurite growth was carried as previously described^62^. DRG neurons were isolated, purified and plated on 96-well PDL coated plates with or without laminin. Compounds were added to the well and DRG neurons fixed after 3 days and stained using a mouse monoclonal antibody against bIII-tubulin (TUJ1; 1:800 Millipore-sigma # T8578) followed by anti-mouse Alexa Fluor 568 (Thermofisher; #A-11004) and Hoechst (thermofisher; #62249) for nuclear staining. Neurite outgrowth was analyzed with an ArrayScan High Content Screening System (Cellomics). Data including neuron counts, neuronal cell body area, neurite total length, neurite maximum length and fluorescence intensity were collected with a neuron selecting algorithm. Pictures were acquired with a 10× objective using the ArrayScan high-resolution camera mode and analyzed based on the “Neuronal profiling” bioapplication.

### Statistics and reproducibility

Comparisons between groups were made using students’ *t*-test and one or two-way ANOVA followed by multiple comparison test, as appropriate. Comparisons of distributions were made using the Kolmogorov-Smirnov test in Stata (StataCorp LP). Quantile-quantile plots were derived in Excel (Microsoft) by interpolation. Statistical analysis was performed using Prism version 10 for Windows, GraphPad Software (La Jolla, CA, USA). All in vitro experiments were carried out at least twice. The sample size was determined when designing the assay (neuron density, capsaicin concentration) and the data is presented in Fig. 1. For anatomical and behavioral results, the sample size was based on literature and previous experience with the techniques used. All experiments were carried out and analyzed blinded to treatment to reduce bias.

### Reporting summary

Further information on research design is available in the Nature Portfolio Reporting Summary linked to this article.

### Supplementary information

Supplementary Information

Description of Additional Supplementary Files

Supplementary Data 1

Reporting Summary

## Data Availability

The datasets generated during and/or analyzed during the current study are available from the corresponding authors on reasonable request. The numerical source data for all graphs in the paper can be found in Supplementary Data 1.
