# Supplementary material for: Bortezomib-induced neuropathy is in part mediated by the sensitization of TRPV1 channels
Source: Commun Biol. 2023 Dec 5;6:1228. doi: 10.1038/s42003-023-05624-1 (PMC10698173; doi:10.1038/s42003-023-05624-1)

Supplementary Information

Supplementary Table 1. Most potent sensitizers of TRPV1 responses following 24 hours of exposure to DRG in vitro model system from the BioMol Molecule set. 35 compounds (excluding HBSS and Capsaicin controls).

COMPOUND	Function	% Sensitization
FCCP	Disrupts mitochondrial calcium buffering	10.31
Tyrphostin 9	mitochondrial fission inducer, PDGFR tyrosine kinase inhibitor	9.26
Hoechst 33342·3HCl (BisBenzimide)	DNA minor groove binder -	9.02
Okadaic acid	Has been shown to increase NGF transcript in astrocytes	8.93
Puromycin·2HCl	Protein synthesis inhibitor	7.90
staurosporine	Non-selective kinase inhibitor	7.42
Gliotoxin	Proteasome inhibitor	7.32
Monensin sodium	Na+ ionophore, blocks intracellular protein transport	6.93
Cycloheximide	Inhibits protein biosynthesis at the translational step	6.79
5-Iodotubercidin	ERK2 inhibitor	6.76
Brefeldin A	Targets Sar1, a GEF. Inhibits protein transport from ER to golgi.	6.72
Ro 31-8220	PKC inhibitor	6.65
Calyculin A	Phosphatase inhibitor	6.58
Actinomycin D	Transcription inhibitor	6.44
LY-83583	reduces cGMP	6.38
Mitomycin C	cross links DNA	6.37
Valinomycin	K+ ionophore - causes mitochondrial swelling	6.18
Beta-lapachone	Topoisomerase I inhibitor	5.91
Doxorubicin	Topoisomerase II inhibitor	5.74
Pepstatin	protease inhibitor	5.63
Alamethicin	Cation ionophore	5.41
EHNA	PDE2 inhibitor, increased cGMP and cAMP	5.29
Ionomycin	Ca2+ ionophore	5.26
Camptothecin	Topoisomerase I inhibitor	5.09
TPEN	heavy metal chelator	5.06
Oligomycin A	ATP synthase inhibitor - disrupts mitochondrial uptake of calcium	5.00

HBDDE	PKC inhibitor	4.80
Ouabain	Na+/K+-aTPase inhibitor	4.36
Parthenolide	reduces HDAC1	4.28
Diphenyleneiodonium	flavoprotein inhibitor	4.25
Vinblastine	tubulin inhibitor	4.09
MG-132	Proteasome inhibitor	4.08
Z-Leu3-VS	Proteasome inhibitor	4.06

Supplementary Table 2. Most potent negative modulators of TRPV1 responses (desensitizers) following 24 hours of exposure to DRG in vitro model system from the BioMol Molecule set. 35 compounds (excluding HBSS and Capsaicin controls) that resulted in lowest calcium responses as described in methods section for Hamamatsu System calcium imaging.

Compound Name	Conc. in DMSO	Final Conc.	Compound description	24A-RelativeAUC	24B-RelativeAUC	AverageAUC
Mead ethanolamide	1mM	20nM	Endocannabinoids: Cannabinoid receptor agonist	-0.1199954	-0.139950895	-0.129973132
Arachidonic acid (20:4, n-6)	1mM	20nM	Bioactive lipids: Polyunsaturated fatty acid	-0.1187229	-0.110958941	-0.114840914
Amiodarone·HCl	5mg/mL	100ng/mL	Ion channel ligands: Calcium channels	-0.1667368	-0.031368283	-0.099052531
Nimodipine	5mg/mL	100ng/mL	Ion channel ligands: Calcium channels	-0.0251714	-0.170313922	-0.097742666
Docosatrienoic acid (22:3 n-3)	1mM	20nM	Bioactive lipids: Polyunsaturated fatty acid	-0.0301393	-0.133821273	-0.08198029
FK-506	5mg/mL	100ng/mL	Inhibitors: FKBP ligand	-0.0824058	-0.076028021	-0.079216934
PAF C18:1	1mM	20nM	Bioactive lipids: PAF receptor agonist	-0.0193667	-0.130942511	-0.075154615
Flecainide acetate	5mg/mL	100ng/mL	Ion channel ligands: Sodium channels	-0.0055875	-0.143129013	-0.074358247
TTNPB	1mM	20nM	Nuclear receptor ligands: Retinoid RAR agonist	-0.0576782	-0.086076609	-0.071877384
15(S)-HETE	0.1mM	2nM	Bioactive lipids: Bioactive arachidonic acid metabolite	-0.0174036	-0.116084585	-0.066744092
Palmitylethanolamide	1mM	20nM	Endocannabinoids: Cannabinoid CB2 receptor agonist	-0.126098	-0.00373263	-0.064915302
Wortmannin	5mg/mL	100ng/mL	Lipid biosynthesis: PI-3Kinase, other kinases inhibitor	-0.1017011	-0.025522559	-0.063611849
L-cis-Diltiazem·HCl	5mg/mL	100ng/mL	Ion channel ligands: Calcium channels	-0.0331601	-0.083742689	-0.058451377
Cypermethrin	5mg/mL	100ng/mL	Inhibitors: calcineurin inhibitor	-0.0423405	-0.067906286	-0.055123412
1-Acyl-PAF	1mM	20nM	Bioactive lipids: PAF agonist	-0.0497559	-0.060097962	-0.054926942
Thalidomide	5mg/mL	100ng/mL	Inhibitors: TNFalpha synthesis inhibitor	-0.0548572	-0.054031201	-0.054444205
K252A	0.5mg/mL	10ng/mL	Kinase inhibitors: Kinase inhibitor (Broad spectrum)	-0.0746006	-0.034006124	-0.054303367
Eicosa-5,8-dienoic acid (20:2 n-12)	1mM	20nM	Bioactive lipids: Polyunsaturated fatty acid	-0.0631662	-0.039267634	-0.051216935
17-Octadecynoic acid	1mM	20nM	Lipid biosynthesis: Inhibits fatty acid omega oxidation	-0.0025163	-0.096649662	-0.049582979
NPPB	5mg/mL	100ng/mL	Ion channel ligands: Misc. channels	-0.0514712	-0.047238415	-0.049354803
Linoleic acid	1mM	20nM	Bioactive lipids: Polyunsaturated fatty acid	-0.0623248	-0.035091696	-0.048708233
Prostaglandin F2a	1mM	20nM	Bioactive lipids: Prostaglandin FP receptor agonist	-0.0910637	-0.006287874	-0.048675799
Yohimbine	5mg/mL	100ng/mL	CNS receptor ligands: Adrenoreceptor antagonist (alpha)	-0.0465708	-0.050706971	-0.048638907
Trequinsin	5mg/mL	100ng/mL	Inhibitors: phosphodiesterase (PDE3) inhibitor	-0.0532915	-0.04291583	-0.048103679
L-erythro-MAPP	1mM	20nM	Lipid biosynthesis: Negative control for D-erythro-MAPP	-0.0007655	-0.094729614	-0.047747534
GM6001	5mg/mL	100ng/mL	Protease inhibitors: broad spectrum MMP inhibitor	-0.0981724	0.006872515	-0.045649967
Dipalmitoylphosphatidic acid	1mM	20nM	Bioactive lipids: Activates MAP kinase cascade	-0.0139678	-0.077102618	-0.045535207
4-hydroxyphenylretinamide	1mM	20nM	Nuclear receptor ligands: Retinoid receptor agonist / apoptosis inducer	-0.1449457	0.054128054	-0.045408846

L-744,832	5mg/mL	100ng/mL	Inhibitors: Ras farnesyltransferase inhibitor	-0.0636194	-0.026508743	-0.045064054
Flunarizine·2HCl	5mg/mL	100ng/mL	Ion channel ligands: Calcium channels	-0.0499443	-0.037998628	-0.043971458
Tyrphostin-8	5mg/mL	100ng/mL	Inhibitors: Calcineurin inhibitor	-0.0442994	-0.043209548	-0.043754464
LY-171883	1mM	20nM	Bioactive lipids: Leukotriene D4 receptor antagonist	0.02810082	-0.115277257	-0.04358822
N-acetyl-S-geranylgeranyl-L-Cysteine	1mM	20nM	Bioactive lipids: ICMT inhibitor	-0.0345662	-0.051291078	-0.042928656
12(S)-HPETE	0.1mM	2nM	Bioactive lipids: Fatty acid hydroperoxide	-0.0598671	-0.025983342	-0.042925222
AM-580	1mM	20nM	Nuclear receptor ligands: Retinoid RAR agonist	-0.0252155	-0.060038996	-0.042627231

Supplementary Figure 1. (a) Complete response percentages among DRG cells for TRPA1 (AITC), TRPV1 (CAP = capsaicin) and KCl (all responsive neurons). *: $p<0.05$, one-way ANOVA followed by Dunnett's multiple comparisons test, $F(2, 6) = 437.4$. (b) Small scale pilot screen performed with peptide ligands and small molecules to identify direct activators of sensory neurons. *: $p<0.05$, one-way ANOVA followed by Dunnett's multiple comparisons test, $F(10, 23) = 19.81$. (c) Basic composition of the ScreenWell® ICCB Known Bioactives Library (BioMol). (d) Twenty ligands in the BioMol library produce robust and significant activation of sensory neurons. Potentiation from control indicates the change (%) of overall signal. Each dot represents a duplicate experiment.

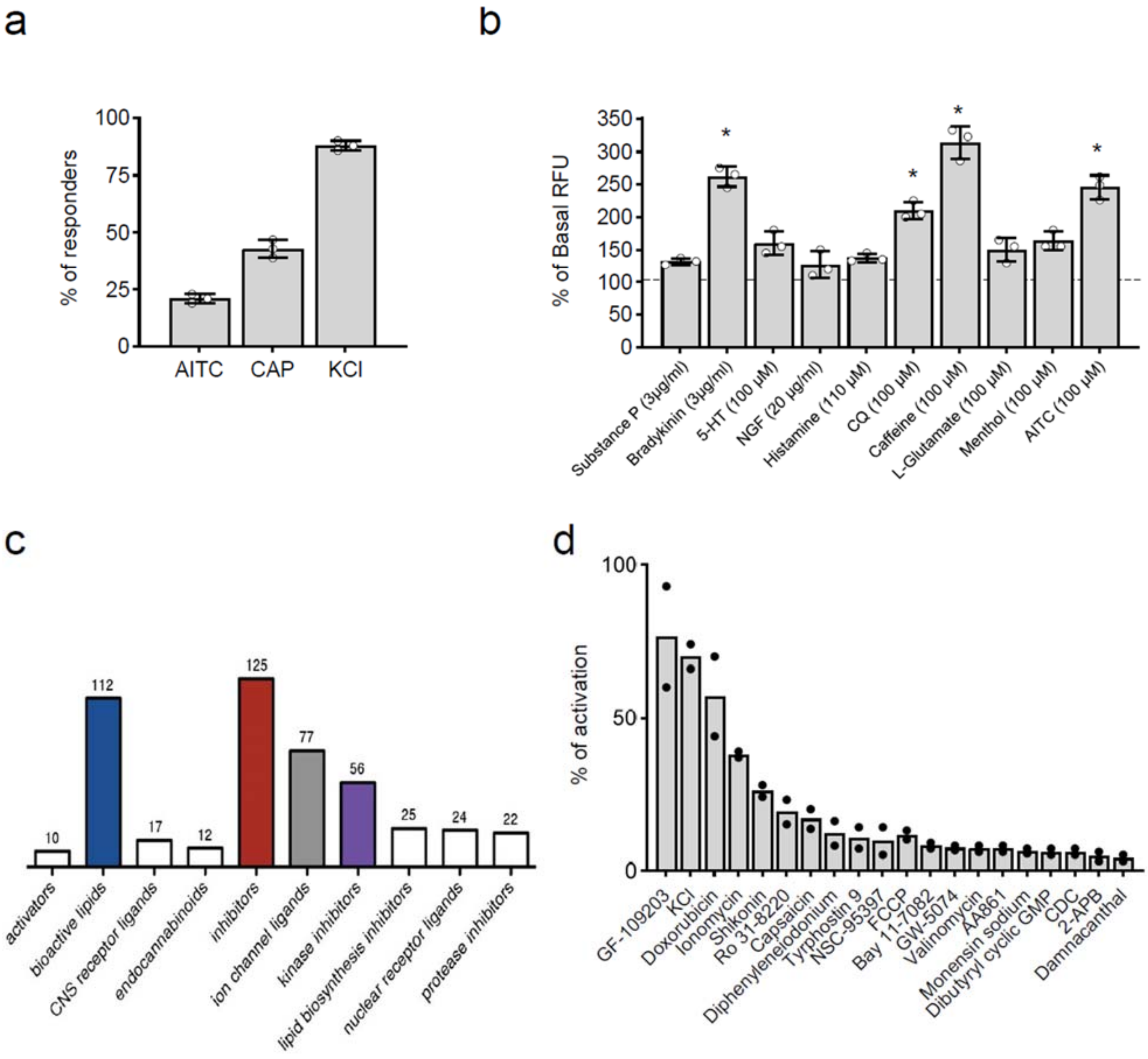

Supplementary Figure 2. TRPV1 mRNA expression levels remain unchanged following treatment with Bortezomib (1uM).

a

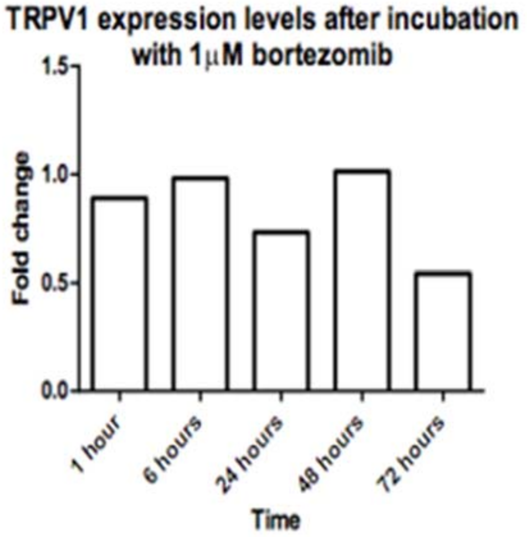

Supplementary Figure 3. (a) Illustration of non-vertical axons (that cross the dermis-epidermis border) and vertical axons (that are only present in the epidermis). (b) IENFD assessed in TRPV1-TdT mice to label TRPV1-lineage negative sensory neurons. Quantification classified vertical and non-vertical axons in the skin section. *: $p<0.05$, Student's T test ($t=2.368$; $df=11$ for non-vertical) (c) Time spent in seconds in different temperature zones during the 90 minutes of the thermal gradient assay for saline- or bortezomib-treated mice at various time points after treatment. (d) Time spent in seconds in different temperature zones during the 90 minutes of the thermal gradient assay for saline- or bortezomib-treated TRPV1 KO mice.

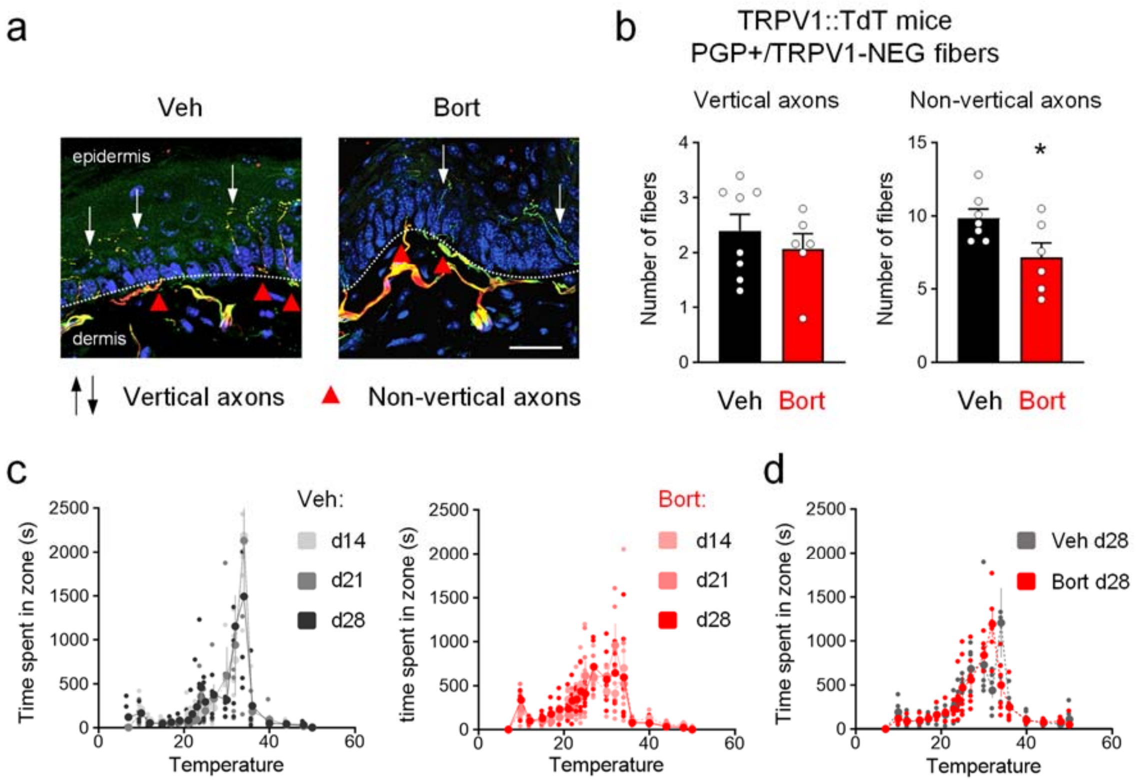

Supplement: Supplementary file 1 — Supplementary Information [file 42003_2023_5624_MOESM1_ESM.pdf]
