# Supplementary material for: Bortezomib-induced neuropathy is in part mediated by the sensitization of TRPV1 channels
Source: Commun Biol. 2023 Dec 5;6:1228. doi: 10.1038/s42003-023-05624-1 (PMC10698173; doi:10.1038/s42003-023-05624-1)
Supplement: Supplementary file 2 — Description of Additional Supplementary Files [file 42003_2023_5624_MOESM2_ESM.pdf]

Description of Additional Supplementary Files

File name: Supplementary Data 1

Description: The source data behind the graphs in the main and supplementary figures.
