# Supplementary material for: Bortezomib-induced neuropathy is in part mediated by the sensitization of TRPV1 channels
Source: Commun Biol. 2023 Dec 5;6:1228. doi: 10.1038/s42003-023-05624-1 (PMC10698173; doi:10.1038/s42003-023-05624-1)
Supplement: Supplementary file 4 — Reporting Summary [file 42003_2023_5624_MOESM4_ESM.pdf]

nature portfolio

Corresponding author(s): Alban Latremoliere & Clifford J Woolf

Last updated by author(s): Nov 18, 2023

Reporting Summary

Nature Portfolio wishes to improve the reproducibility of the work that we publish. This form provides structure for consistency and transparency in reporting. For further information on Nature Portfolio policies, see our Editorial Policies and the Editorial Policy Checklist.

Statistics

For all statistical analyses, confirm that the following items are present in the figure legend, table legend, main text, or Methods section.

n/a	Confirmed	
☐	☒	The exact sample size (*n*) for each experimental group/condition, given as a discrete number and unit of measurement
☐	☒	A statement on whether measurements were taken from distinct samples or whether the same sample was measured repeatedly
☐	☒	The statistical test(s) used AND whether they are one- or two-sided<br>*Only common tests should be described solely by name; describe more complex techniques in the Methods section.*
☐	☒	A description of all covariates tested
☐	☒	A description of any assumptions or corrections, such as tests of normality and adjustment for multiple comparisons
☐	☒	A full description of the statistical parameters including central tendency (e.g. means) or other basic estimates (e.g. regression coefficient) AND variation (e.g. standard deviation) or associated estimates of uncertainty (e.g. confidence intervals)
☐	☒	For null hypothesis testing, the test statistic (e.g. *F*, *t*, *r*) with confidence intervals, effect sizes, degrees of freedom and *P* value noted<br>*Give P values as exact values whenever suitable.*
☒	☐	For Bayesian analysis, information on the choice of priors and Markov chain Monte Carlo settings
☒	☐	For hierarchical and complex designs, identification of the appropriate level for tests and full reporting of outcomes
☐	☒	Estimates of effect sizes (e.g. Cohen's *d*, Pearson's *r*), indicating how they were calculated

Our web collection on statistics for biologists contains articles on many of the points above.

Software and code

Policy information about availability of computer code

Data collection	Cells were imaged in Hamamatsu FDSS7000Ex imaging apparatus with a CCD camera C9100-13. Data were collected and organized in Microsoft Excel. Additionally, figures were plotted on Graphpad Prism analysis and visualization software.<br><br>Calcium imaging was also conducted on a Nikon Ti Eclipse inverted microscope.<br><br>Axopatch 200A amplifier from Molecular Devices was used to collect data and digitized with a Digidata 1440A A/D interface and recorded using pCLAMP 10 software from Molecular Devices.
Data analysis	Data for Hamamatsu experiments were analyzed in Microsoft Excel. Additional calcium imaging experiments were analyzed in NIS Elements software (AR 3.10), and further analysis was conducted using Microsoft Excel. Statistical analyses were carried out oh Graphpad PRISM 9.

For manuscripts utilizing custom algorithms or software that are central to the research but not yet described in published literature, software must be made available to editors and reviewers. We strongly encourage code deposition in a community repository (e.g. GitHub). See the Nature Portfolio guidelines for submitting code & software for further information.

Data

Policy information about availability of data

All manuscripts must include a data availability statement. This statement should provide the following information, where applicable:

- Accession codes, unique identifiers, or web links for publicly available datasets
- A description of any restrictions on data availability
- For clinical datasets or third party data, please ensure that the statement adheres to our policy

The datasets generated during and/or analyzed during the current study are available from the corresponding authors on reasonable request

Research involving human participants, their data, or biological material

Policy information about studies with human participants or human data. See also policy information about sex, gender (identity/presentation), and sexual orientation and race, ethnicity and racism.

Reporting on sex and gender	N/A
Reporting on race, ethnicity, or other socially relevant groupings	N/A
Population characteristics	N/A
Recruitment	N/A
Ethics oversight	N/A

Note that full information on the approval of the study protocol must also be provided in the manuscript.

Field-specific reporting

Please select the one below that is the best fit for your research. If you are not sure, read the appropriate sections before making your selection.

☒ Life sciences ☐ Behavioural & social sciences ☐ Ecological, evolutionary & environmental sciences

For a reference copy of the document with all sections, see nature.com/documents/nr-reporting-summary-flat.pdf

Life sciences study design

All studies must disclose on these points even when the disclosure is negative.

Sample size	Statistical methods were not used to predetermine sample size. The size of the cohort, based on similar studies in the field, was validated by pilot studies. All sample sizes are indicated in the figures and/or figure legends. All n values are indicated within the figure legends. For in vitro high-throughput experiments, we included at least three biological replicates.
Data exclusions	No data were removed from the calcium imaging experiments. For in vivo experiments, animals would be excluded for health-related issues as stated in IACUC protocols. For immunostaining experiments, only slides with adequate tissue quality were included in analyses.
Replication	Replication is accounted for in measuring means and standard errors in imaging. In Q-Q plots, two different populations of cells were compared to each other, with the smaller population data being interpolated to match corresponding quantile. In that image, each dot represented a virtual representative of the population. In example traces of calcium imaging, each line was a responsive cell within a population of cells. The data presented in 2B are illustrative samples. In vitro neurite growth experiments and in vivo experiments were duplicated at least twice.
Randomization	In the in vitro experiments using imaging, cells were drawn from a common pool of primary sensory DRG neurons. The location of the compounds within the 384-well plates remained consistent throughout the experimentation due to logistical challenges - representing a lack of randomization. For in vivo work, randomization was achieved by assigning random numbers to animals for treatment. Each cage had animals treated with vehicle or bortezomib.
Blinding	Experimenters were blinded fat the time of experiments. For neurite growth assays, nerve density assessments and behavioral analyses, the experimenters were blinded at the time of analysis and the code was lifted once all samples were processed.

Reporting for specific materials, systems and methods

We require information from authors about some types of materials, experimental systems and methods used in many studies. Here, indicate whether each material, system or method listed is relevant to your study. If you are not sure if a list item applies to your research, read the appropriate section before selecting a response.

Materials & experimental systems

n/a	Involved in the study
☐	☒ Antibodies
☒	☐ Eukaryotic cell lines
☒	☐ Palaeontology and archaeology
☐	☒ Animals and other organisms
☒	☐ Clinical data
☒	☐ Dual use research of concern
☒	☐ Plants

Methods

n/a	Involved in the study
☒	☐ ChIP-seq
☒	☐ Flow cytometry
☒	☐ MRI-based neuroimaging

Antibodies

Antibodies used	PGP 9.5 primary antibody (1:1000 Millipore-sigma # AB5898; TUJ1; 1:800 Millipore-sigma # T8578
Validation	Doran et al. (1983) J. Neurochem 40 1542-1547, Omura et al. 2016 Neuron 4;90(3):662

Animals and other research organisms

Policy information about studies involving animals; ARRIVE guidelines recommended for reporting animal research, and Sex and Gender in Research

Laboratory animals	Male adult C57Bl/6j mice (JAX#000664) and mice lacking TRPV1 (JAX#003770).
Wild animals	This study did not involve wild animals.
Reporting on sex	This study was conducted in male adult mice.
Field-collected samples	This study did not involve field-collected samples.
Ethics oversight	All procedures were approved by the Boston Children's Hospital's Institutional Animal Care and Use Committee.

Note that full information on the approval of the study protocol must also be provided in the manuscript.
